# *In vitro* studies on the pharmacological potential, anti-tumor, antimicrobial, and acetylcholinesterase inhibitory activity of marine-derived *Bacillus velezensis* AG6 exopolysaccharide†

**DOI:** 10.1039/d3ra04009g

**Published:** 2023-09-04

**Authors:** Maha A. Alharbi, Amani A. Alrehaili, Mona Othman I. Albureikan, Amal F. Gharib, Hussam Daghistani, Maha M. Bakhuraysah, Ghfren S. Aloraini, Mohammed A. Bazuhair, Hayaa M. Alhuthali, Ahmed Ghareeb

**Affiliations:** a Department of Biology, College of Science, Princess Nourah bint Abdulrahman University P.O. Box 84428 Riyadh 11671 Saudi Arabia maalharbi@pnu.edu.sa; b Department of Clinical Laboratory Sciences, College of Applied Medical Sciences, Taif University P.O. Box 11099 Taif 21944 Saudi Arabia Arhili@tu.edu.sa maha1@tu.edu.sa amgharib@tu.edu.sa hmhuthali@tu.edu.sa; c Department of Biological Sciences, Faculty of Science, King Abdulaziz University Jeddah 21589 Saudi Arabia malboraikan@kau.edu.sa; d Department of Clinical Biochemistry, Faculty of Medicine, King Abdulaziz University Jeddah 21589 Saudi Arabia hmdaghistani@kau.edu.sa; e Regenerative Medicine Unit, King Fahd Medical Research Center, King Abdulaziz University Jeddah 21589 Saudi Arabia; f Department of Medical Laboratory Sciences, College of Applied Medical Sciences, Prince Sattam bin Abdulaziz University Al-Kharj 11942 Saudi Arabia g.aloraini@psau.edu.sa; g Department of Clinical Pharmacology, Faculty of Medicine, King Abdulaziz University Jeddah 21589 Saudi Arabia obazohair@kau.edu.sa; h Botany and Microbiology Department, Faculty of Science, Suez Canal University Ismailia 41522 Egypt aghareeb@science.suez.edu.eg

## Abstract

In the current study, *Bacillus velezensis* AG6 was isolated from sediment samples in the Red Sea, identified by traditional microbiological techniques and phylogenetic 16S rRNA sequences. Among eight isolates screened for exopolysaccharide (EPS) production, the R6 isolate was the highest producer with a significant fraction of EPS (EPSF6, 5.79 g L^−1^). The EPSF6 molecule was found to have a molecular weight (Mw) of 2.7 × 10^4^ g mol^−1^ and a number average (Mn) of 2.6 × 10^4^ g mol^−1^ when it was analyzed using GPC. The FTIR spectrum indicated no sulfate but uronic acid (43.8%). According to HPLC, the EPSF6 fraction’s monosaccharides were xylose, galactose, and galacturonic acid in a molar ratio of 2.0 : 0.5 : 2.0. DPPH, H_2_O_2_, and ABTS tests assessed EPSF6’s antioxidant capabilities at 100, 300, 500, 1000, and 1500 μg mL^−1^ for 15, 60, 45, and 60 minutes. The overall antioxidant activities were dose- and time-dependently increased, and improved by increasing concentrations from 100 to 1500 μg mL^−1^ after 60 minutes and found to be 91.34 ± 1.1%, 80.20 ± 1.4% and 75.28 ± 1.1% respectively. Next, EPSF6 displayed considerable inhibitory activity toward the proliferation of six cancerous cell lines. Anti-inflammatory tests were performed using lipoxygenase (5-LOX) and cyclooxygenase (COX-2). An MTP turbidity assay method was applied to show the ability of EPSF6 to inhibit Gram-positive bacteria, Gram-negative bacteria, and antibiofilm formation. Together, this study sheds light on the potential pharmacological applications of a secondary metabolite produced by marine *Bacillus velezensis* AG6. Its expected impact on human health will increase as more research and studies are conducted globally.

## Abstract 2

In the current study, *Bacillus velezensis* AG6 was isolated from sediment samples in the Red Sea, identified by traditional microbiological techniques and phylogenetic 16S rRNA sequences.

## Graphical abstract

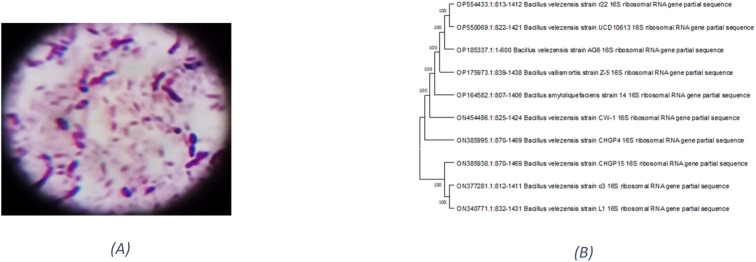

## 1. Introduction

Exopolysaccharides (EPSs) are carbohydrate polymers with a high molecular weight that surround most microbial cells in the marine environment.^1^ They constitute a significant percentage of the ocean’s reduced carbon reservoir and enable marine bacteria to survive by modulating the physicochemical environment close to the bacterial cell.^2^ Marine polysaccharides can be categorized into various groups based on their origins, including marine animals, plants, and microbial polysaccharides, each displaying their distinctive structure.^3^

Microbial EPSs are organic, miscible, or immiscible polysaccharides synthesized by microorganisms and released into the extracellular medium. They adhere to the cell’s surface in broth media.^4^ EPSs may be produced by a broad range of microorganisms, some examples of which include bacteria, cyanobacteria, fungi, and yeasts. Microbial EPSs help cells survive by attaching to surfaces, aggregating, and preventing desiccation.^5^ Also, a gelled polysaccharide layer around the cell may affect its diffusion properties, both into and out of the cell.^6^ Additionally, they help the microbial communities survive changes in temperature, saltiness, and nutrient unavailability.^7^

Marine microbial polysaccharides exhibit a variety of structures and unique properties, especially those produced extracellularly. In microbial polysaccharides, glucose, galactose, and mannose are the most prevalent monosaccharides, making up most of these heteropolysaccharides.^8^ Marine microbial polysaccharides include glucuronic acid, galacturonic acid, amino sugars, pyruvate, sulfates, and uronic acids, unlike terrestrial plant polysaccharides.^9^

Most EPS are linear and have high molecular weights (1–3 × 10^5^ Da). The combination of pyruvate and uronic acid linked to ketals, along with inorganic residues such as sulfate or phosphate, is primarily responsible for the bulk of reported EPS being polyanionic.^10^

Due to the growing need for natural polymers in industries such as food and pharmaceuticals, there has been a recent surge in interest in polysaccharides produced by microorganisms.^11^ There is an increasing curiosity in discovering and identifying new polysaccharides from microorganisms that may have potential uses as anti-inflammatory, antioxidant, antimicrobial, anticytotoxic agents, and many other pharmacological applications.^12–16^ For example, A hetero acidic EPS produced by the isolated *Bacillus cereus* strain AG3 from Red Sea sediments inhibited the growth of methicillin-resistant *Staphylococcus aureus* (MRSA) and displayed the potential to represent a new class of anti-inflammatory drug.^13^ Asker *et al.*^17^ found that the *Achromobacter piechaudii* NRC2 EPS fraction has substantial anti-cyclooxygenase and antioxidant activities. Liu *et al.* isolated two polysaccharides from the fermenting fluid of *Floccularia luteovirens* that showed free radical scavenging activities.^18^ Additionally, Sulfated exopolysaccharide (levan) derived from *Bacillus megaterium* PFY-147 was identified by Pei *et al.* The substance demonstrated notable antioxidant and probiotic properties, indicating its potential efficacy in biomedical applications.^19^

The World Health Organization (WHO) estimates that cancer was the main cause of 9.5 million deaths globally in 2020. According to estimates, 17 million people will die from cancer by 2040 due to a growing incidence of the disease. These statistics highlight the pressing need for new and improved therapies.^20^

Surgery, radiation, chemotherapy, and immunotherapy all have drawbacks. Due to tumor size, site, stage, and metastasis, cancer treatment is complicated. Such therapies generally fail to control tumors due to resistance, and various side effects occur during or after treatment.^21,22^ Therapeutic microorganisms may overcome some these limitations of traditional cancer treatments. Bacteria alone can be effective anticancer agents, and they can be genetically modified to generate and release specific chemicals and tailor their metabolic pathways. Therapeutic microorganisms also penetrate tumor tissue and target hypoxic regions of tumors. Another application is as a carrier for delivering tumoricidal and immunotherapeutic drugs. Since then, and even today, many investigators have reported that specific live, attenuated, and modified microbes, including *Clostridium*, *Bifidobacterium*, *Salmonella*, *Mycobacterium*, *Bacillus*, and *Listeria*, have the capacity to target cancer cells specifically and function as anticancer agents.^23^

Deepak *et al.* documented anti-tumor activity of microbial EPS, where the EPS from *Lactobacillus acidophilus* showed *in vitro* effect on colon cancer cell lines.^24^ Also, Wang *et al.* reported the anticancer effects of an EPS from a newly isolated *B. breve* strain against head and neck squamous cell carcinoma cell lines.^25^ EPSR3 from marine *Bacillus cereus* was reported to have a cytotoxicity-inhibiting effect on the growth of T-24, MCF-7, and PC-3 carcinoma cell lines.^13^

Therefore, based on the remarkable ESP applicability and ongoing attempts to explore and investigate novel exopolysaccharides. This investigation extracted and characterised a new EPS from the marine *Bacillus velezensis* strain AG6 from the Red Sea sediments. Furthermore, the EPSF6 compound was tested *in vitro* to evaluate its potential as an antioxidant, anticancer, anti-inflammatory, antimicrobial, antibiofilm, and anti-acetylcholine esterase inhibitor.

## 2. Materials and methods

### 2.1. Sample collection and bacterial isolation

Red Sea sediments were obtained, and the serial dilution technique was carried out to isolate the bacteria from the collected samples.^26^

### 2.2. Bacterial isolates identification

The bacterial strains were selected based on physiochemical properties, their distinctive cultural characteristics, and the highest production rate of EPSs.^27^ For molecular identification, phylogenic analysis was conducted.^28^

The BLAST tool was employed to compare the obtained DNA sequence to the GenBank database at the NCBI. This was followed by an alignment to assess the resemblance between the isolate’s sequence and those in the database.

### 2.3. EPS production and fractionation

*Bacillus velezensis* AG6 was chosen for the significant production of EPS. The fermentation medium’s broth was followed by C. Liu *et al.*^29^ Four liters of chilled ethanol were added to the supernatant to facilitate fractional precipitation. Between 200 and 800 nanometers, UV absorption spectra were analyzed to detect whether proteins and nucleic acids were present.^30^

### 2.4. EPS chemical analysis

EPSF6-FTIR spectra were acquired using potassium bromide (KBr) pellets. A total of 2.0 mg of sample was added to 200 mg KBr and the mixture was applied to the FTIR-UNIT Bruker Vector 22 Spectrophotometer in Coventry, UK.^31^ The average molecular weight (Mw) of EPSF6 was measured by High-performance Gel Permeation chromatography (HPGPC, Agilent 1100 Series System, Hewlett-Packard, Germany). Water was used as the eluent solvent at a flow rate of 0.5 mL min^−1^ over a GEL GMPWXL column. Refractive index (RI) detection was used to measure the average molecular weight (Mw). The Mw/Mn ratio was used to construct the polydispersity index (PI).

The presence of uronic acid in EPSF6 samples was detected using a colorimetric method described by Filisetti-Cozzi and Carpita. The method involved diluting the sample with concentrated sulfuric acid(2 mL), boiling the mixture for 20 minutes at 100 °C, cooling it to room temperature, and then adding *m*-hydroxydiphenyl (150 μL). The absorbance of the resulting mixture was measured at 520 nm after an hour.^32^ The amount of sulfate in EPSF6 was determined using the Garrido’s method. Five milligrams of EPSF6 was hydrolyzed in a sealed tube with 5 mL of formic acid (88%) at 105 °C for 5 hours. After dryness, In a 100 milliliter measuring flask, 10 mg of BaCl_2_ was dissolved in a small quantity of H_2_0. 20 mL of Tween 20 was added, and the final volume was adjusted to 100 mL with distilled H_2_0. To 10 milliliters of the hydrolysate solution, 1 mL of dilute hydrochloric acid (0.3 N) and 1 mL of the BaCl_2_-Tween 20 reagent was added. After mixing, the solution was allowed to stand for 15 minutes and then mixed again. The optical density of the mixture was then read at 500 nm against a blank containing distilled water instead of the sulfate solution.^33^ The method outlined by Randall *et al.* were used to determine the monosaccharide quantity EPSF6 was hydrolyzed with 2 M trifluoroacetic acid at 120 °C for 2 hours.^34^ The resulting mixture was diluted with methanol and dried under vacuum. The residue was then dissolved in water and analyzed on an Aminex carbohydrate HP-87C column(300 × 7.8 mm) using water as the eluent and a flow rate of 0.5 mL min^−1^(Agilent 1100 Series System, Santa Clara, CA, USA). The standards used were mannose (Man), glucose (Glc), d-glucuronic acid (GlcA), galactose (Gal), and d-galacturonic acid (GalA).

UV absorption spectra between 200 and 800 nanometers were examined for proteins and nucleic acids.^30^

### 2.5. Antioxidant assessment of the EPS

#### 2.5.1. Antioxidant DPPH assay

To examine the antioxidant capacity of the EPS, a methodology developed based on the approach described by Brand-Williams *et al.* was used.^35^

#### 2.5.2. Hydrogen peroxide scavenging (H_2_O_2_) assay

The ability of the EPS to remove H_2_O_2_ was assessed following Ruch *et al.*^36^

#### 2.5.3. Antioxidant ABTS˙^+^ assay

According to the methodology outlined by Miller *et al.*,^37^ the capacity of EPS to scavenge ABTS radical cations was examined at a range of concentrations, including 100, 300, 500, 1000, and 1500 μg mL^−1^.

### 2.6. Cytotoxic evaluation of the EPS on different cell lines

Different cell lines human liver cancer cell line (HepG2), adenocarcinoma human alveolar basal epithelial cells (A-459), human colon cancer cell line (HCT-116), human breast cancer cell line (MCF-7), human epithelioma-2 (Hep-2) and PC-3 (human prostate carcinoma cells) were brought from the American-type culture collection (ATCC, Rockville, MD). The cells were cultured using RPMI-1640 media, which was supplemented with 10% activated fetal bovine serum and 50 μg mL^−1^ gentamycin. The cells were incubated at a temperature of 37 °C with a carbon dioxide concentration of 5%. Cell lines were treated with various doses of EPS (0, 31.25, 62.5, 125, 250, 500, 1000, 2000, and 4000 μg mL^−1^) and their viability was assessed to determine its cytotoxicity.^38^

### 2.7. Anti-inflammatory assessment

#### 2.7.1. *In Vitro* lipoxygenase (LOX) inhibition

The EPS’s inhibitory effect on the 5-LOX enzyme was determined using the method described by Granica *et al.*^39^

#### 2.7.2. *In Vitro* cyclooxygenase (COX-2) inhibition

EPS’s effectiveness at reducing inflammation was measured by comparing its ability to inhibit the COX-2 enzyme to the reference drug Celecoxib.^40,41^

### 2.8. Antibacterial and antibiofilm activity

The antibacterial and antibiofilm activity of EPS was tested. These test organisms included Gram-positive bacteria (*Staphylococcus aureus* NRRLB-767 and *Bacillus subtilis* ATCC 6633), Gram-negative bacteria (*Escherichia coli* ATCC 25922, *Klebsiella pneumoniae* ATCC 10145), yeast (*Candida albicans* ATCC 10231) and fungi (*Aspergillus niger* NRRLA-326) were used as test organisms.^42^ The control in this test was the pathogen without any treatment.

The microtiter plate assay (MTP) method was utilized to examine the ability of EPS to inhibit biofilm formation against (*Staphylococcus aureus* NRRLB-767 and *Escherichia coli* ATCC-25922).^43^

### 2.9. Acetylcholine esterase inhibitory effect

The inhibitory effect of EPS was tested using Abcam kits (Biomedical Campus, CB2 0AX, Cambridge, UK) according to the method described by Monserrat *et al.*^44^

### 2.10. Statistical analysis

**Scheme 1:**
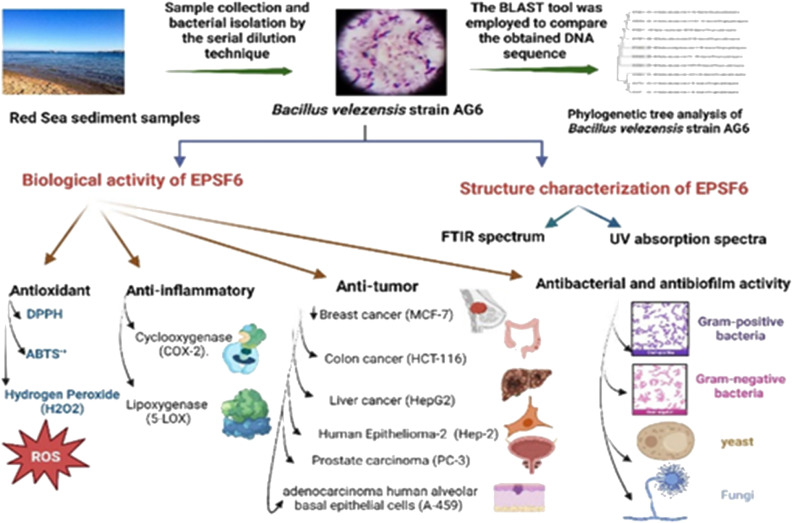
Flow chart displaying production, isolation, and purification of exopolysaccharide (EPSF6).

The data were analyzed by ANOVA one-way for multiple comparisons Scheme 1. The Kolmogorov–Smirnova and Shapiro–Wilk tests verified that our data were regularly distributed. To assess the similarity between various concentrations, Duncan’s test was used. using the IBM-SPSS statistics program (version 25) at *P* ≤ 0.05, and a t-test (*n* = 3 replicates) was used in comparisons.

## 3. Results

### 3.1. Screening, isolation, and identification of bacterial isolates

**Fig. 1:**
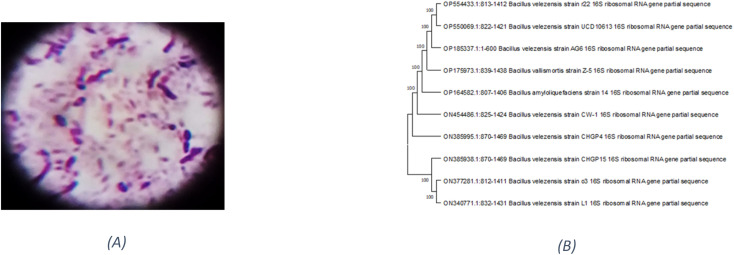
(A) Gram +ve stain of *Bacillus velezensis* strain AG6. (B) Phylogenetic tree analysis of *Bacillus velezensis* strain AG6 according to 16S rRNA gene sequencing demonstrating representative species of the genus *Bacillus*.

Based on their colony morphology, eight marine sediment bacterial isolates were selected for screening to create EPSs. The greatest EPS produced by one of these marine bacteria was (F6), which was isolated from the Red Sea. AG6 strain has the highest EPS production among the marine bacteria identified as having a substantial EPS yield, with a production of (5.79 g L^−1^). The isolated strain AG6’s morphological, physiological, and biochemical tests revealed it to be a Gram-positive rod (Fig. 1A) large irregular colony, pale yellow, rough colony texture, dull colony surface, convex, flat elevation, non-capsulated, spore-producing, non-motile, and non-acid fast (Table S1†). Catalase, Voges–Proskauer, Simon citrate, urease, and nitrate reduction assays were positive. While coagulase, Indol, Methyl Red, oxidase, and H_2_S tests returned negative (Table S2†). The phylogenetic tree was produced by comparing sequences highly similar to the rRNA sequences of the target bacterium. The resultant rRNA gene sequences were found to correspond to the *Bacillus velezensis* (Fig. 1B), which led to the conclusion that the tree was generated correctly. The nucleotide sequence data of the isolated bacterial strain was searched against the GenBank database. The identification of *Bacillus velezensis* AG6 was confirmed with accession number (OP185337.1). The BLAST tool was used for analyzing the submitted DNA sequence and submitting the NCBI GenBank database.

### 3.2. Production and chemical composition analysis of EPSF6

Exopolysaccharide (EPSF6) with a yield of 5.79 g L^−1^ was generated from the R6 bacterial strain. The crude residue obtained was then purified through fractionation and precipitation methods. EPSF6 was treated with deionized water for three days, after which it was filtered through a membrane with a 100-micron pore size. Cold ethanol was gradually added to the dialyzed sample, causing fractional precipitation. After this process, the EPSF6 core fraction (89.8%) was obtained by performing three ethanol precipitation steps on the crude EPS.

**Fig. 2:**
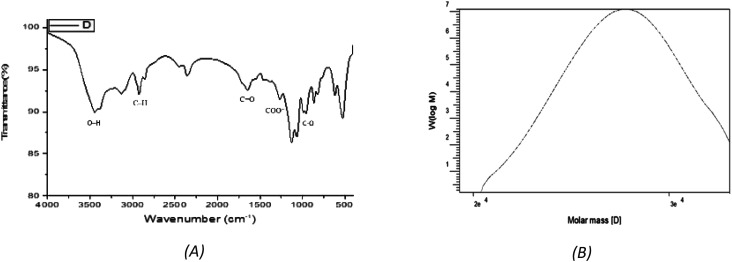
(A) FTIR spectrum of EPSF6 showing the main functional groups. (B) GPC analysis of EPSF6.

The Uronic acid (43.8%) but no sulfate was in EPSF6. These acidic fractions are xylose, galactose, and galacturonic acid monosaccharides, with molar ratios of 2.0 : 0.5 : 2.0 (Fig. S1†). EPSF6 molecules in the GPC chromatogram were widely scattered (Fig. 2B) with a polydispersity index (PI) of 1.1, revealed (Mw) of 2.7 × 10^4^ g mole^−1^, and (Mn) of 2.6 × 10^4^ g mole^−1^.

The stretching vibration of O–H in sugar residue components caused the FTIR spectra fraction to peak at 3443.28 cm^−1^. Circular vibrations also produced a band at 1647.87 cm^−1^. The band at 864.917 cm^−1^ disrupted the C–O glycosidic bond’s stretching vibration. The band at 863.953 cm^−1^ showed pyranose ring vibrations (Fig. 2A).

### 3.3. Antioxidant assessment of EPSF6

**Fig. 3:**
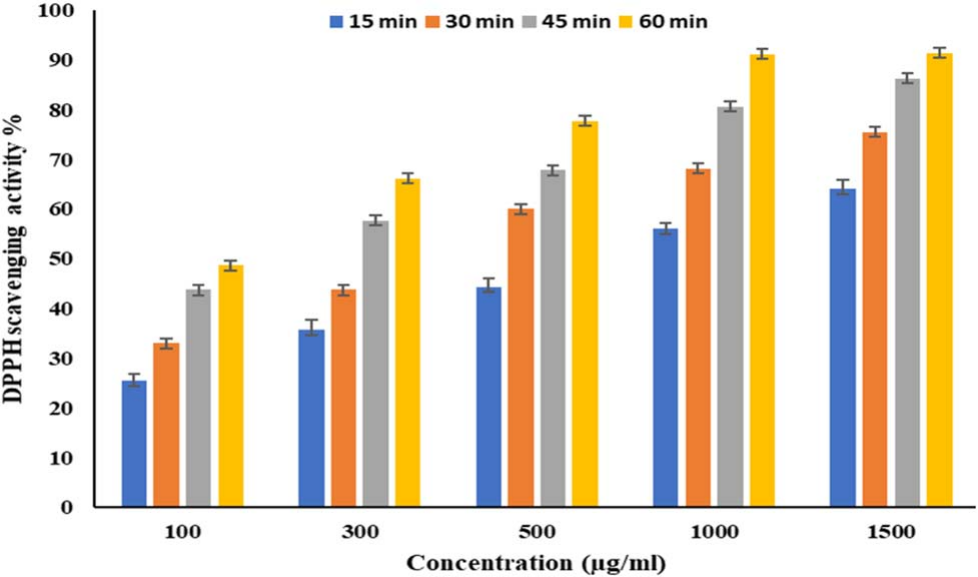
The scavenging activity of EPSF6 against the DPPH radicals at different concentrations and time intervals. Data are presented as mean ± SD. ANOVA one-way was used for data analysis (*n* = 3, *P* < 0.05).

**Fig. 4:**
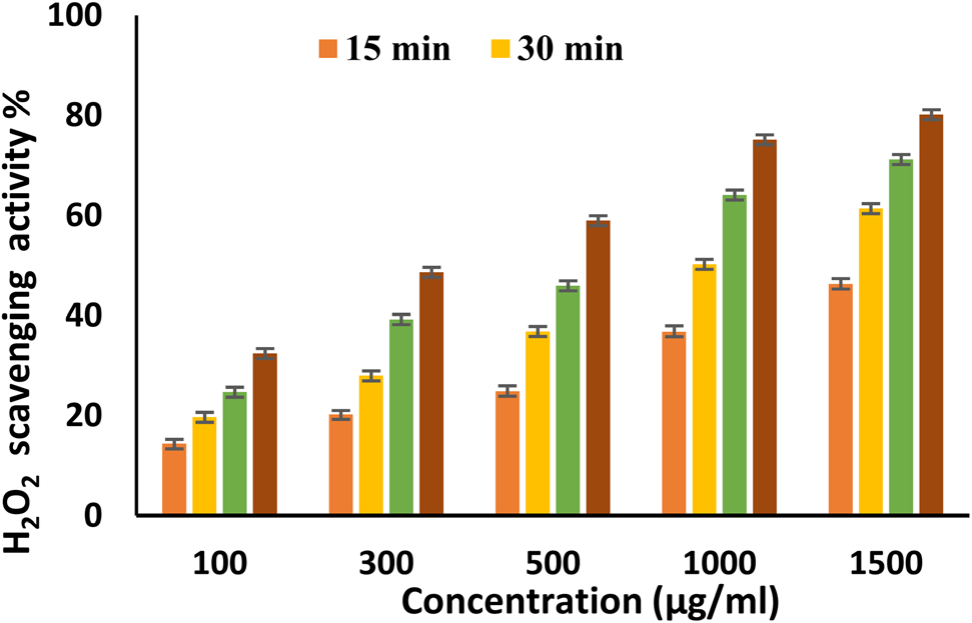
H_2_O_2_ scavenging activity of EPSF6 at different concentrations and time intervals. Data are presented as mean ± SD. ANOVA one-way was used for data analysis (*n* = 3, *P* < 0.05).

**Fig. 5:**
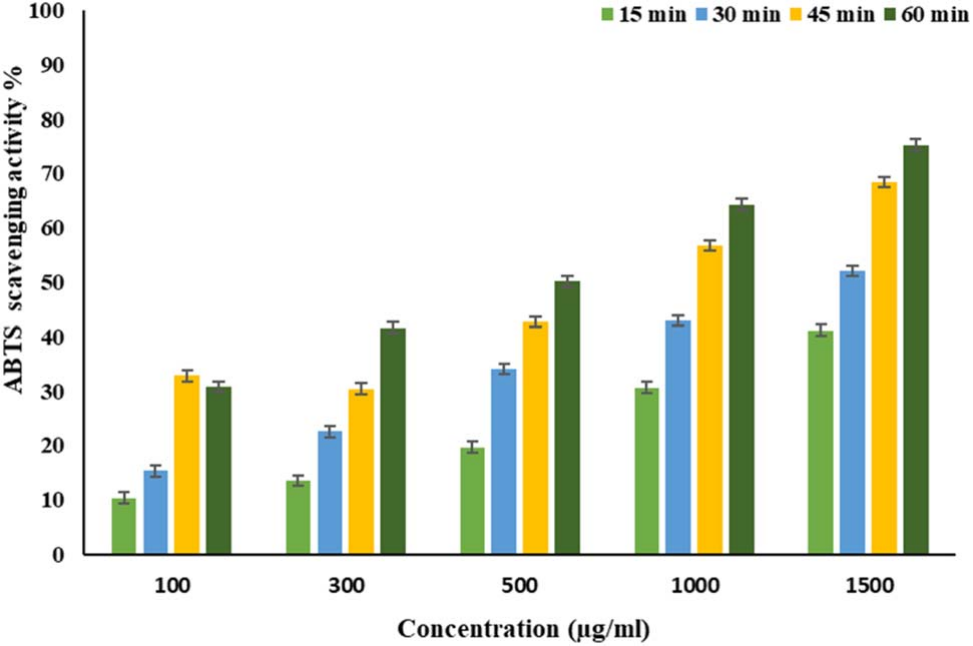
EPSF6 scavenging activity against ABTS at different concentrations and time intervals. Data are presented as mean ± SD. ANOVA one-way was used for data analysis (*n* = 3, *P* < 0.05).

EPSF6 was tested for its ability to scavenge DPPH radicals at 100, 300, 500, 1000, and 1500 μg mL^−1^ doses for 15, 60, 45, and 60 minutes. The overall antioxidant activity is improved by increasing EPSF6 concentrations from 100 to 1500 μg mL^−1^. After 60 minutes, the highest level of antioxidant activity was 91.34 ± 1.1% at 1500 μg mL^−1^ (Fig. 3). Therefore, after 60 minutes, the IC_50_ against the DPPH radical was around 100 μg mL^−1^ compared to the IC_50_ of control = 86.44 ± 1.42 μg mL^−1^ (Table S3†). The maximum extreme activity was 80.20 ± 1.4% at 1500 μg mL^−1^ after 60 minutes of testing EPSF6's ability to scavenge H_2_O_2_ at various concentrations (100, 300, 500, 1000, and 1500 μg mL^−1^). After 60 minutes, the IC_50_ value for the H_2_O_2_ radical was around 300 μg mL^−1^ (Fig. 4) compared to the IC_50_ of the control = 88.71 ± 0.98 μg mL^−1^ (Table S3†). The maximum extreme activity measured for ABTS scavenging activity was 75.281.1% at 1500 μg mL^−1^ after 60 min (Fig. 5). EPSF6’s scavenging activity was tested at 100, 300, 500, 1000, and 1500 μg mL^−1^. The control’s IC_50_ was 87.50 ± 0.75 μg mL^−1^, whereas the ABTS radicals were 500 μg mL^−1^ after 60 minutes (Table S3†).

### 3.4. Anti-tumor evaluation of EPSF6

**Fig. 6:**
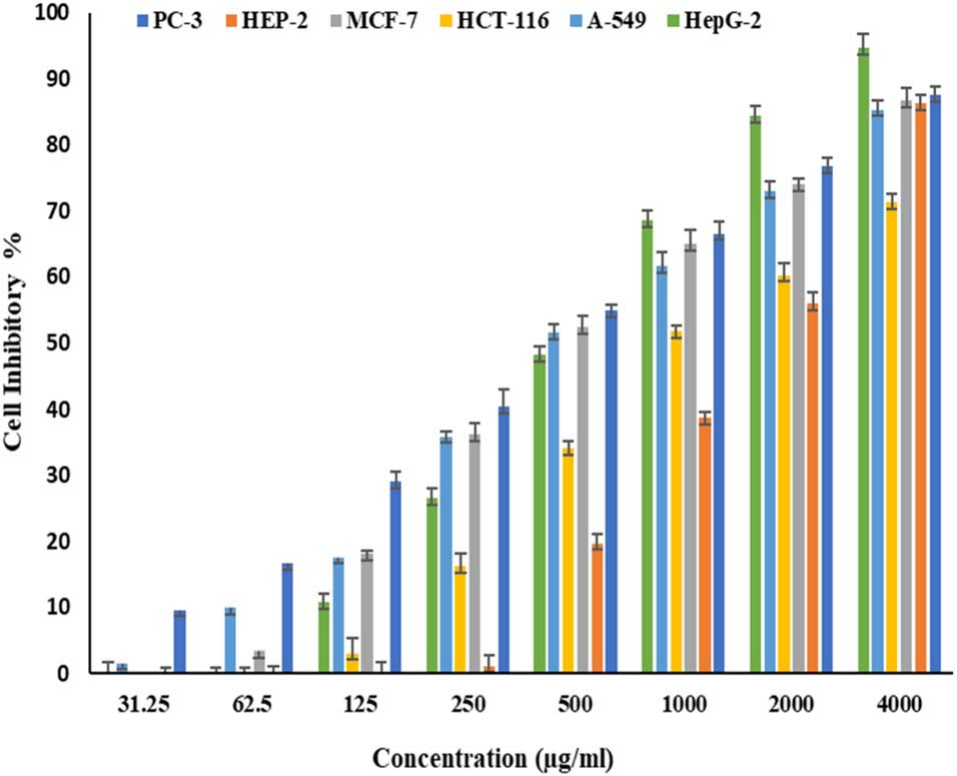
Cytotoxicity activity of different concentrations of EPSF6 on % inhibitory of different cancer cell lines. Data represent mean ± SD of triplicate measurements.

**Fig. 7:**
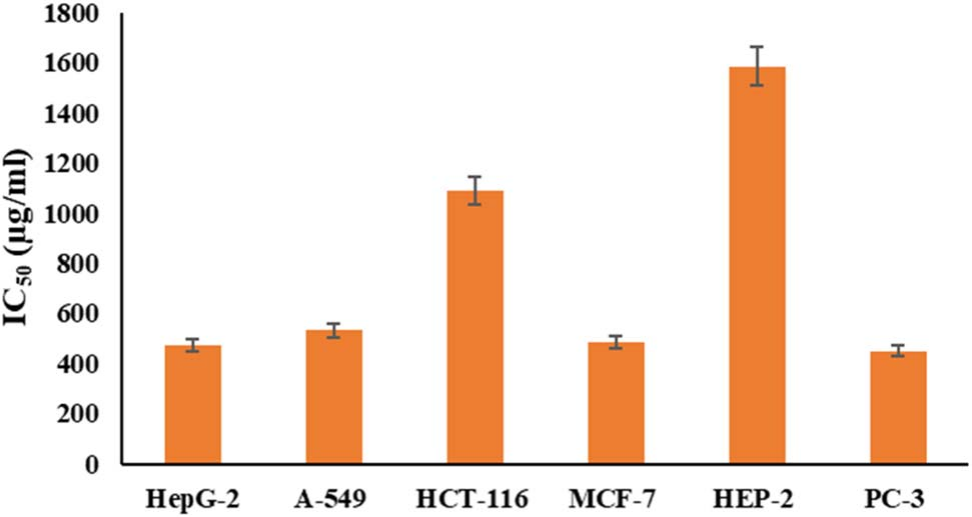
IC_50_ of EPSF6 on % viability of the tested cancer cell lines. The data is presented as the mean ± SD of three measurements.

Fig. 6 and 7 show the effect of different concentrations of EPSF6 on the survival rate of several types of cells, including HepG-2, A-549, HCT-116, MCF-7, HEP-2, and PC-3. The highest and lowest IC_50_ of EPSF6 for cell lines were reported for HEP-2 and PC-3 as 1586.22 ± 14.8 and 450.45 ± 12.1 μg mL^−1^, respectively, compared to Cisplatin IC_50_ (4.21, 1.29 μg mL^−1^) (Table S4†).

**Fig. 8:**
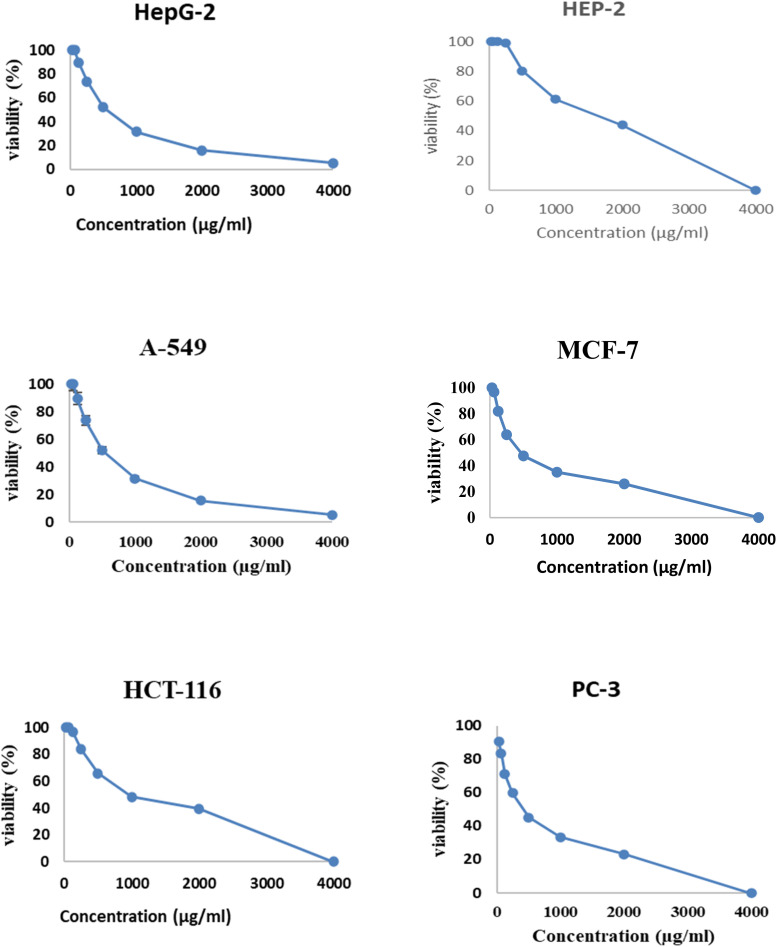
Analysis of EPSF6’s effect on cell viability % in cell lines at different doses.

These cell lines' respective IC_50s_ for EPSF6 were (471.88 ± 15.2 μg mL^−1^, 532.81 ± 12.5 μg mL^−1^, 1.089 ± 21.58 μg mL^−1^, 483.54 ± 19.82 μg mL^−1^, 1586.22 ± 14.8 μg mL^−1^ and 450.45 ± 12.1 μg mL^−1^). As the concentration of EPSF6 decreased in the cell lines examined, the percentage of viable cells increased. At 125 μg EPSF6 per mL concentrations and above, the percentage of viable cells in most cell lines began to drop significantly compared to the control cells. This decline continued as the concentration increased (Fig. 8).

### 3.5. Anti-inflammatory activity of EPSF6

**Fig. 9:**
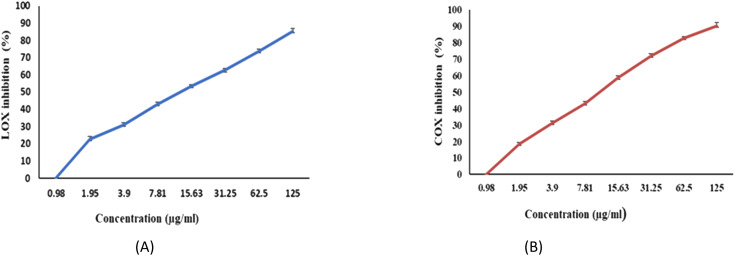
Anti-Inflammatory activity of EPSF6 using different methods (A) 5-LOX (B) COX-2. Data are presented as mean ± SD. ANOVA one-way was used for data analysis (*n* = 3, *P* < 0.05).

The ability of EPSF6 to reduce inflammation was evaluated by analyzing the degree to which the cyclooxygenase (COX-2) and lipoxygenase (5-LOX) were stimulated or in-hibited. Compared to ibuprofen IC_50_ = 1.5 ± 1.3 g mL^−1^ for 5-LOX and celecoxib IC_50_ = 0.28 ± 1.7 g mL^−1^ for COX-2, the average IC_50_ value for EPSF6 on 5-LOX and COX-2 was 14.21 ± 1.20 and 16.82 ± 1.01 g mL^−1^, respectively (Fig. 9A and B). As the concentration of EPSF6 was raised, it was found that the degree to which it inhibited 5-LOX and COX-2 activities rose in a dose-dependent manner.

### 3.6. Antimicrobial and anti-biofilm assessment of EPSF6

The antimicrobial effects of EPSF6 were evaluated against the growth of two-Gram positive and two-Gram negative bacteria, a yeast, and one fungus. EPSF6 exhibited antimicrobial activity for Gram +ve rather than Gram −ve bacteria. EPSF6 reduced the growth of *B. subtilis* by 34% compared to Ciprofloxacin 97%, and *E. coli* by 19% compared to 98% of ciprofloxacin with non-significant anti-candidal activity by 7%, nor anti-fungal activity by 5% comparing to Nystatine 97%, 98% respectively (Table 1).

**Table 1:** MTP antimicrobial assay of EPSF6 against different microorganisms

Compounds	Antimicrobial activity (%)					
	Gram positive		Gram negative		Yeast	Fungi
	*S. aureus* NRRLB-767	*B. Subtilis* ATCC 6633	*E. Coli* ATCC 25922	*K. pneumoniae* ATCC 10145	*C. albicans* ATCC 10231	*Aspergillus niger* NRRLA-326
EPSF6	27.32 ± 0.75	34.19 ± 0.91	19.05 ± 0.61	13.64 ± 0.66	7.58 ± 0.17	5.92 ± 0.41
Ciprofloxacin	96.01 ± 0.43	97.24 ± 0.18	98.07 ± 0.35	98.10 ± 0.27	—	—
Nystatine	—	—	—	—	97.16 ± 0.90	98.23 ± 0.16

Further, EPSF6 antibiofilm activity was tested by the microtiter plate assay (MTP) method against two bacterial strains (*Staphylococcus aureus* NRRLB-767 and *Escherichia coli* ATCC-25922), with no significant antibiofilm inhibition for either of the tested bacteria 35.673 ± 0.79 and 16.932 ± 098 respectively (Table 2).

**Table 2:** Antibiofilm inhibition of EPSF6 towards *E. coli* and *S.aureus*

	Biofilm inhibition ratio (%)	
	*E. Coli* ATCC 25922	*S. aureus* NRRLB-767
EPSF6	16.932 ± 098	35.673 ± 0.79

### 3.7. Evaluation of AChE activity of EPSF6

**Fig. 10:**
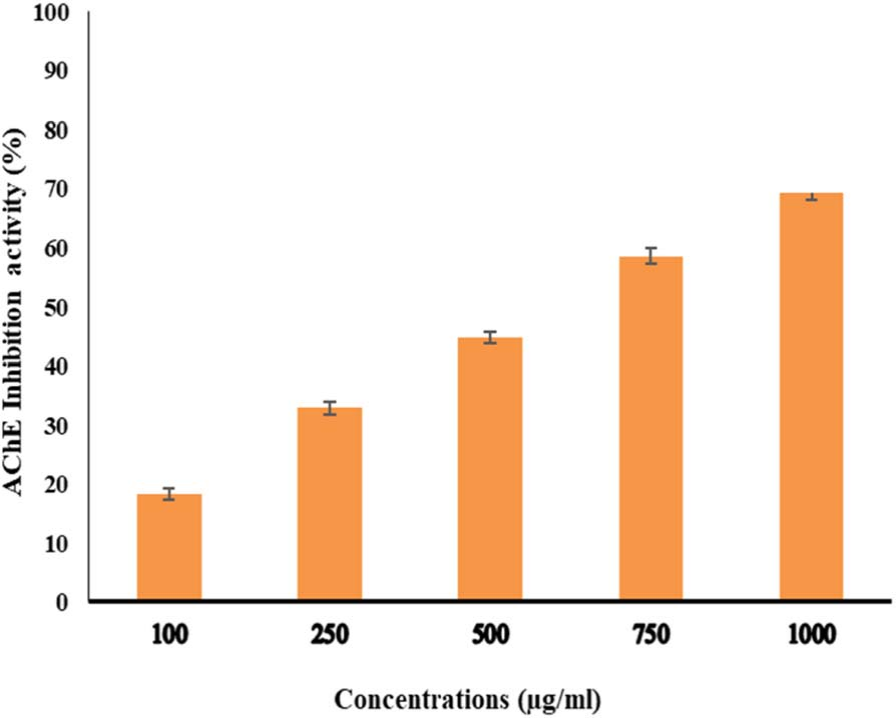
Acetylcholine esterase inhibition activity by different concentrations of EPSF6. Data presented as mean ± SD. ANOVA one-way was used for data analysis (*n* = 3, *P* < 0.05).

The activity of AchE was compared between two compounds, EPSF6 and Eserine, which served as a control, by measuring it at different concentrations ranging from 0.02, 0.04, 0.06, 0.08, 0.1, and 0.12 μg mL^−1^. The average IC_50_ of EPSF6 was 439.05 μg mL^−1^ (Fig. 10), which was much higher than that of Eserine (0.09 μg mL^−1^) (Table S7†). When the concentration of the EPSF6 fraction was raised from 100 to 1000 μg mL^−1^, there was a significant decrease in the activity of AChE, implying that EPSF6 had a more suppressive effect on the enzyme than eserine.

## 4. Discussion

Over the past ten years, the number of approved anticancer medications for clinical use has steadily risen.^45^ Despite these advances, drug efficacy, toxicity, and pricing challenges still need to be solved. These issues are particularly problematic in undeveloped countries since pharmaceuticals are relatively scarce.^46^ Therefore, the search for natural remedies is growing through efforts to find alternative therapeutics leveraging microbial species. Bacterial secondary metabolites continue to make a substantial and varied impact on contemporary medical treatments. With more global research and studies, their influence on human health is expected to expand.^47^

The EPS explored was derived from a spore-forming, Gram-positive, non-capsulated marine *Bacillus velezensis* strain AG6 (accession no.: OP185337.1) (Fig. 1). Of the eight strains of bacteria studied, the F6 strain was identified as the most significant producer of EPS (EPSF6). The EPSF6 weighed 5.79 g L^−1^ with a main fraction of 89.8% (three-volume ethanol). The chemical analysis of EPSF6 revealed a (Mw) of 2.7 × 10^4^ g mol^−1^ and a (Mn) of 2.6 × 10^4^ g mol^−1^ comprised of xylose, galactose, and galacturonic acid with a molar ratio 2.0 : 0.5 : 2.0 respectively. Also, there was no sulfate present but 43.8% uronic acid, which signifies that it is an acidic polysaccharide (Fig. 2B and 1S†). As previously stated, EPSF6 has a high (Mw) of 2.7 × 10^4^ g mol^−1^. Most marine exopolysaccharides are composed of linear chains of mono sugars. On average, the molecular weight ranges from 1 × 10^5^ Da to 3 × 10^5^ Da^10^. Even though the vast majority of EPS polymers are neutral, the vast majority are polyanionic because they include uronic acid. One example of this is EPSF6.

Moving to EPSF6 antioxidant investigation by DPPH, H_2_O_2_, and ABTS assays, the maximum antioxidant activates were (91.34 ± 1.1, 80.20 ± 1.4, and 75.28 ± 1.1%). The antioxidant activity increased with increased tested concentration (Fig. 3–5). It is important to note that glutathione, a potent non-enzymatic antioxidant, is synthesized with the help of secreting enzymes like superoxide dismutase, which can be related to free radical scavenging ability.^48^

Additionally, various side chemical groups, such as the sulfated, hydroxyl, and uronic acid groups, promote the scavenging of antioxidant.^49^ The explored EPSF6 by FTIR revealed it has no sulfate but uronic acid (43.8%) (Fig. 2B).

An EPS derived from *Bacillus albus* DM-15, obtained from ayurvedic treatment in India, has a notable effect on scavenging the activity of three different radicals: DPPH (58.1%), ABTS (70.4%), and NO (58.9%) depending on the concentration., which is consistent with our findings.^50^ Also, *B. cereus* strain AG3 was reported with a peak antioxidant capacity of 90.4 ± 1.6% at 1500 μg mL^−1^ after approximately 2 hours and an IC_50_ of around 500 μg mL^−1^ after 1 hour when tested against the DPPH radical. Also, it was observed that at 1500 μg mL^−1^, the scavenging activity of H_2_O_2_ was 75% after 60 minutes and the IC_50_ was reported to be around 1500 μg mL^−1^ after 15 minutes.^13^ Additionally, from marine *Pseudomonas* PF-6, Ye *et al.*^51^ identified and purified an acidic β-type EPS that exhibited antioxidant action. Additionally, it was shown that an EPS isolated from *Bacillus amyloliquefaciens* 3 MS 2017 could scavenge DPPH free radicals with a maximal activity of 99.39% at a concentration of 1000 μg mL^−1^.^52^ As well, *Streptomyces carpaticus* produced an EPS with DPPH antioxidant potential with an IC_50_ value of 111 μg mL^−1^.^53^

The reductive ability of such monosaccharides may cause EPS’s ability to scavenge radicals.^54^ In several studies, purified polysaccharides derived from crude polysaccharides were found to be more functional *in vitro* than crude polysaccharides.^55,56^ Compositionally, the chemical analysis of EPSF6 by HPLC revealed three different monosaccharides, xylose, galactose, and galacturonic acid, with molar ratios of 2.0 : 0.5 : 2.0, respectively (Fig. S1†). These monosaccharides, with the exception of glucuronic acid, are powerful reductive agents due to the presence of an aldehyde group in their structures.

After that, the MTT assay was used to investigate the cytotoxic potential of EPSF6 in six different cell lines. These cell lines' respective IC_50s_ for EPSF6 were (471.88 ± 15.2, 532.81 ± 12.5, 1.089 ± 21.58, 483.54 ± 19.82, 1586.22 ± 14.8 and 450.45 ± 12.1 μg mL^−1^), respectively (Fig. 8) where control Cisplatin IC_50_ against same cancerous cell lines were (1.29, 4.08, 2.36, 3.41, 4.21, 3.79 μg mL^−1^) respectively (Table S4†).

In line with our findings, recent research investigated whether or not EPSR5 isolated from marine *Kocuria* sp. had a suppressive impact on the growth of cancer cells.^12^ The highest IC_50s_ was (1691.00 ± 44.20 μg mL^−1^) for MCF-7, and the lowest was (453.46 ± 21.80 μg mL^−1^) for HepG-2. An exopolysaccharide from *Bacillus albus* DM-15 isolated from Indian Ayurvedic had an IC_50_ value of 20 ± 0.97 μg mL^−1^ against lung cancer cell line (A549), and cellular staining showed necrotic, apoptotic properties in damaged A549 cells.^50^

Additionally, a new strain of *Bacillus subtilis* generated an acidic EPSR4 that displayed notable antiproliferative effects on the HepG-2, A-549, and T-24 cell lines.^57^ He *et al.*, 2015 examined the anti-tumor effects of an exopolysaccharide LEP-2b from *Lachnum* YM405 on hepatic, colon, and lung cell lines after modifying and adding sulfates and phosphates to the EPSs. They found an increase in their cytotoxic activity.^58^ Moreover, The EPSs from strains of *P. aeruginosa* were found to be cytotoxic against HT-29 cells with IC_50_ values at 44.8 (EPS-A) and 12.7 (EPS-B) μg mL^−1^, which renders them as natural and effective anticancer drugs.^59^

Among EPS-generating species, *L. helveticus*, *L. acidophilus*, and *L. plantarum* produced the most frequently associated EPS with promising anticancer potential.^55^ Even within the same species, EPS’s ability to inhibit proliferation varied from strain to strain.^60^ EPS has been reported to influence or obstruct the activity of genes involved in carcinogenesis, including p53, BCL2, and many others.^61^ Moreover, the antiproliferative properties of EPS may be explained by the presence of distinctive structures such as uronic acid and sulfate.^62^ In our explored polymer, EPSF6 contained no sulfate but uronic acid (43.8%) in contrast to our findings, (EPSR4) from the marine *Bacillus subtilis* isolated from the Red Sea and found it sulfated (48.2%) and had no uronic acid.^57^ However, as mentioned earlier, the chemical composition of EPS varies from one habitat to another and from species to another, and even within the same species.^4^

Moving to investigate the anti-inflammatory influence of EPSF6 by evaluating its inhibitory impact on 5-LOX and COX-2. Following our findings, EPSR3 isolated from marine *Bacillus cereus* had Lipoxygenase (LOX) inhibitory more potent than the control Ibuprofen and the COX-2 inhibitory compared to Celecoxib.^13^ Also, The anti-inflammatory effectiveness of EPS fractions produced by polluted soil bacteria has been studied by Gangalla and colleagues. Compared to the indomethacin drug, it had significant anti-inflammatory effects (65 ± 0.14, 61 ± 0.15 μg mL^−1^).^63^

Microbial metabolites cause activated macrophages to produce pro-inflammatory cytokines TNF-, IL-1, IL-6, and IL10, as well as other cytokines and transcription factors connected to them.^64^ For example, TNF-α and interleukins 12, 15, and 18 were observed to be downregulated by peptides extracted from *Yersinia pestis*.^65^ Its structure and cyclooxygenase inhibition effect is thought to be responsible for this anti-inflammatory property.^31^ Also, it was reported that a lipopeptide produced by *Bacillus liceniformis* VS16 increased IL-10 and TGF and decreased TNF-α and IL Iβ.^66^ Further, EPSF6 was tested by MTP plate assay antimicrobial and antibiofilm agent against two G +ve, two G −ve bacteria, *C. albicans* ATCC 10231 and *A. niger* NRRLA-326, but neither activity was significantly considered (Tables 1 and 2).

The AChE enzyme is highly found in the brain, nerve cells, and RBCs, and it is involved in hydrolyzing the acetylcholine ester.^67^ In some neurological illnesses, the activity of the acetylcholinesterase (AChE) enzyme and other cholinergic system enzymes is decreased. The amyloid deposition has been linked to the etiology of Alzheimer’s disease and CNS neuronal impairment. Where the metabolism of beta-amyloid precursor has been attributed to cholinergic hyperactivity,^68^ given its effects on beta-amyloid metabolism, there is a potential for AChE inhibitors to be used as a clinical neuroprotective therapy for neurological disorders like senile dementia, ataxia, myasthenia gravis, and Alzheimer’s disease.^69–71^

By preventing Ach hydrolysis, altering the AChE activity may help to restore the cholinergic balance, slow the progression of Alzheimer’s disease, and improve cognition. Finding new AChE inhibitors for therapeutic use remains challenging and complex, though, due to problems with gastrointestinal function absorption and bioavailability.^72^

Interestingly, secondary metabolites produced by marine fungi are now found to have neuroprotective properties.^73^ Additionally, research using animal models revealed that COX-2’s inhibitory action lowers inflammation, which is essential for the progression of the neurodegeneration associated with Alzheimer’s disease.^74^ Consequently, several studies have highlighted the potential therapeutic use of non-steroidal COX-2 inhibitors to delay the advancement of Alzheimer’s disease.^75^ Therefore, for such purpose and as another step forward to *in vitro* test EPSF6 anti-AChE activity. EPSF6 was tested at different concentrations (100–1000 μg mL^−1^) with IC_50_ = 439.05 (Fig. 10) compared to IC_50_ Eserine control = 0.09 (μg mL^−1^) (Table S7†).

Accordingly, EPSR4, a compound from the bacteria *Bacillus subtilis*, exhibited a dose-dependent and moderate restraining effect towards AChE action when its IC_50_ was compared to Eserine’s of 0.09 μg mL^−1^, which had an IC_50_ of 786.38 μg mL^−1^.^57^ Also, EPSR5 from marine *Kocuria* sp. yielded IC_50_ = 797.02 compared to Eserine’s IC_50_ = 0.09 μg mL^−1^.^12^ Furthermore, Gangalla *et al.*, 2021 reported the anti-Alzheimer effect of a polysaccharide derived from *Bacillus amyloliquefaciens* RK3 in mice which can be a potential basis for the treatment of many diseases which are characterized by a deficiency in acetylcholine, such as Alzheimer and myasthenia gravis.^76^*Streptomyces lateritius*, or *Streptomyces* sp. UTMC 1334 produces pyrroles and other AChE inhibitors.^77^

It is important to mention that the Astrocytes protect the nervous system against oxidative damage driven by the generation of ROS. Myxobacterial extracts protect human primary astrocytes from oxidative stress.^78^ Myxobacterial extracts from *Archangium* sp. UTMC 4070 and *Cystobacter* sp. UTMC 4073 pretreatments with astrocytes increased brain glutathione, an antioxidant protein complex.^79^ To this end, and because of its specific anti-cyclooxygenase properties, capacity to inhibit acetylcholine esterase, and antioxidant properties, EPSF6 extracted from *Bacillus velezensis* strain AG6 from the Red Sea sediments could be a promising natural heteropolysaccharide for treating or preventing Alzheimer’s disease.

There have been fewer investigations on marine microorganisms' EPS production and recovery but more on its industrial uses. The scarcity of EPSs is due to the limited amount obtained during extraction. A more efficient method for obtaining EPSs, particularly for their synthesis, is needed to increase the availability of EPSs.

Due to the growing demand for EPSs due to their biocompatibility, biodegradability, and non-toxicity, researchers are mixing them with other natural and synthetic polymers to create novel EPSs with new applications in many sectors.^80^ More research is required to ascertain the precise chemical composition and the molecular formula of EPSF6, as well as to determine its biocompatibility *in vivo*, its mode of action, and whether it can alter the composition of the gut microbiome and finally modify them by adding sulfates or phosphate groups to yield derivatives which are more potent and more selective is highly recommended.

## 5. Conclusions

Our study advances the therapeutic utilization of marine bacterial products as abundant sources of bioactive compounds, including pharmaceutically microbial exopolysaccharides. Our work isolated and characterized a novel acidic exopolysaccharide EPSF6 from *Bacillus velezensis* strain AG6 from Red Sea sediments. When tested using a DPPH, H_2_O_2_, ABTS scavenging activity for the explored of EPSF6, which showed significant antioxidant activity, further evidence that EPSF6 is a potent inhibitor of the 5-LOX and COX-2 enzymes points to EPSF6 as a potential anti-inflammatory drug is provided by its substantial inhibitory activity toward both of these enzymes. Continuing our investigation on anticancer activity, EPSF6 significantly inhibits the PC-3 cell line from proliferating. EPSF6 testing for antimicrobial and antibiofilm was non-significant. Finally, our analysis revealed that EPSF6’s capacity to target AChE activity is potentially valuable as a natural treatment for Alzheimer’s. To understand and clarify the reported biological activity of *Bacillus velezensis* strain AG6’s metabolites, future studies should analyze EPSF6’s chemical makeup and chemically modify it by adding sulfates or phosphate groups to produce more potent and selective derivatives. These results demonstrate the possible viability of *Bacillus velezensis* strain AG6 and its future use in the health industry.

## Data Availability

The data presented in this study are openly available in DDBJ/EMBL/GenBank nucleotide sequence databases at https://www.ncbi.nlm.nih.gov, accessed on (10 August 2022), reference number GenBank: OP185337.1.

## Author Contributions

Conceptualization, M. A., A. G. and M. O. I. A.; methodology, A. G., M. A., G. S. A., A. A. A., A. F. G. and M. O. I. A.; software, A. F. G., M. M. B., M. A., A. A. A. and H. M. A; validation, H. M. A., A. A. A. and M. A; formal analysis, G. S. A., H. M. A., M. O. I. A., O. A., A. F. G. and A. G.; investigation, A. G., M. A., M. O. I. A., H. M. A., A. F. G. and M. M. B.; resources, A. G.; data curation, A. A. A, M. M. B. and A. G.; writing—original draft preparation, G. S. A., M. A. and A. G; writing—review and editing, H. D., M. A. B., A. G. and G. S. A.; visualization, A. A. A., A. F. G., M. M. B. and M. O. I. A; supervision, A. G. and M. A.; project administration, M. A.; funding acquisition, M. A. All authors have read and agreed to the published version of the manuscript.

## Conflicts of interest

The authors declare no conflict of interest.

## Supplementary Material

RA-013-D3RA04009G-s001

## References

[cit1] Poli A., Anzelmo G., Nicolaus B. (2010). Mar. Drugs.

[cit2] Dave S. R., Upadhyay K. H., Vaishnav A. M., Tipre D. R. (2020). Environ. Sustainability.

[cit3] Mohammed A. S. A., Naveed M., Jost N. (2021). J. Polym. Environ..

[cit4] Decho A. W., Gutierrez T. (2017). Front. Microbiol..

[cit5] de Carvalho C. C. C. R. (2018). Front. Mar. Sci..

[cit6] Pham J. V., Yilma M. A., Feliz A., Majid M. T., Maffetone N., Walker J. R., Kim E., Cho H. J., Reynolds J. M., Song M. C., Park S. R., Yoon Y. J. (2019). Front. Microbiol..

[cit7] Casillo A., Lanzetta R., Parrilli M., Corsaro M. M. (2018). Mar. Drugs.

[cit8] Yang L., Zhang L.-M. (2009). Carbohydr. Polym..

[cit9] Costa O. Y. A., Raaijmakers J. M., Kuramae E. E. (2018). Front. Microbiol..

[cit10] Xie J.-H., Xie M.-Y., Nie S.-P., Shen M.-Y., Wang Y.-X., Li C. (2010). Food Chem..

[cit11] Albuquerque P. B. S., Coelho L. C. B. B., Teixeira J. A., Carneiro-da-Cunha M. G., Albuquerque P. B. S., Coelho L. C. B. B., Teixeira J. A., Carneiro-da-Cunha M. G. (2016). AIMS Mol. Sci..

[cit12] Alshawwa S. Z., Alshallash K. S., Ghareeb A., Elazzazy A. M., Sharaf M., Alharthi A., Abdelgawad F. E., El-Hossary D., Jaremko M., Emwas A.-H., Helmy Y. A. (2022). Life.

[cit13] Selim S., Almuhayawi M. S., Alharbi M. T., Nagshabandi M. K., Alanazi A., Warrad M., Hagagy N., Ghareeb A., Ali A. S. (2022). Metabolites.

[cit14] Abdel-Wahab B. A., Abd El-Kareem H. F., Alzamami A., Fahmy C. A., Elesawy B. H., Mostafa Mahmoud M., Ghareeb A., El Askary A., Abo Nahas H. H., Attallah N. G. M., Altwaijry N., Saied E. M. (2022). Metabolites.

[cit15] Wang W., Wang S.-X., Guan H.-S. (2012). Mar. Drugs.

[cit16] Wu J., Zhang Y., Ye L., Wang C. (2021). Carbohydr. Polym..

[cit17] Asker M., Mahmoud M., Ibrahim A., Mohamed S. S. (2015). Pharm. Lett..

[cit18] Liu Z., Jiao Y., Hongyun L., Shu X., Chen Q. (2019). Carbohydr. Polym..

[cit19] Pei F., Ma Y., Chen X., Liu H. (2020). Int. J. Biol. Macromol..

[cit20] Sung H., Ferlay J., Siegel R. L., Laversanne M., Soerjomataram I., Jemal A., Bray F., Cancer C. A. (2021). J. Clin..

[cit21] Anand U., Dey A., Chandel A. K. S., Sanyal R., Mishra A., Pandey D. K., De Falco V., Upadhyay A., Kandimalla R., Chaudhary A., Dhanjal J. K., Dewanjee S., Vallamkondu J., Pérez de la Lastra J. M. (2023). Genes Dis..

[cit22] Chakraborty S., Rahman T. (2012). Ecancermedicalscience.

[cit23] Bernardes N., Seruca R., Chakrabarty A. M., Fialho A. M. (2010). Bioeng. Bugs.

[cit24] Deepak V., Ramachandran S., Balahmar R. M., Pandian S. R. K., Sivasubramaniam S. D., Nellaiah H., Sundar K. (2016). In Vitro Cell. Dev. Biol. Anim..

[cit25] Wang L., Wang Y., Li Q., Tian K., Xu L., Liu G., Guo C. (2019). Front. Microbiol..

[cit26] Hayakawa M., Nonomura H. (1987). J. Ferment. Technol..

[cit27] Krieg N. R. , Staley J. T., Brown D. R., Hedlund B. P., Paster B. J., Ward N. L., Ludwig W. and Whitman W. B., Bergey's Manual® of Systematic Bacteriology, Springer New York, New York, NY, 2010

[cit28] Tamura K., Peterson D., Peterson N., Stecher G., Nei M., Kumar S. (2011). Mol. Biol. Evol..

[cit29] Liu C., Lu J., Lu L., Liu Y., Wang F., Xiao M. (2010). Bioresour. Technol..

[cit30] Wang H., Jiang X., Mu H., Liang X., Guan H. (2007). Microbiol. Res..

[cit31] Nicely W. B. , in Advances in Carbohydrate Chemistry, edn. M. L. Wolfrom and R. S. Tipson, Academic Press, 1957, vol. 12, pp. 13–33

[cit32] Filisetti-Cozzi T. M., Carpita N. C. (1991). Anal. Biochem..

[cit33] Garrido M. L. (1964). Analyst.

[cit34] Randall R. C., Phillips G. O., Williams P. A. (1989). Food Hydrocolloids.

[cit35] Brand-Williams W., Cuvelier M. E., Berset C. (1995). LWT–Food Sci. Technol..

[cit36] Ruch R. J., Crist K. A., Klaunig J. E. (1989). Toxicol. Appl. Pharmacol..

[cit37] Miller N. J., Rice-Evans C. A. (1997). Food Chem..

[cit38] Mosmann T. (1983). J. Immunol. Methods.

[cit39] Granica S., Czerwińska M. E., Piwowarski J. P., Ziaja M., Kiss A. K. (2013). J. Agric. Food Chem..

[cit40] Amessis-Ouchemoukh N., Madani K., Falé P. L. V., Serralheiro M. L., Araújo M. E. M. (2014). Ind. Crops Prod..

[cit41] Petrovic N., Murray M. (2010). Methods Mol. Biol..

[cit42] Ingebrigtsen R. A., Hansen E., Andersen J.
H., Eilertsen H. C. (2016). J. Appl. Phycol..

[cit43] Antunes A. L. S., Trentin D. S., Bonfanti J. W., Pinto C. C. F., Perez L. R. R., Macedo A. J., Barth A. L. (2010). APMIS Acta Pathol. Microbiol. Immunol. Scand..

[cit44] Monserrat J., Bianchini A. (2001). Environ. Toxicol. Pharmacol..

[cit45] Sun J., Wei Q., Zhou Y., Wang J., Liu Q., Xu H. (2017). BMC Syst. Biol..

[cit46] Taylor D. , in Pharmaceuticals in the Environment, ed. R. E. Hester and R. M. Harrison, The Royal Society of Chemistry, 2015

[cit47] Abdelghani Z., Hourani N., Zaidan Z., Dbaibo G., Mrad M., Hage-Sleiman R. (2021). Arch. Microbiol..

[cit48] Kurutas E. B. (2016). Nutr. J..

[cit49] Wang J., Hu S., Nie S., Yu Q., Xie M. (2015). Oxid. Med. Cell. Longevity.

[cit50] Vinothkanna A., Sathiyanarayanan G., Rai A. K., Mathivanan K., Saravanan K., Sudharsan K., Kalimuthu P., Ma Y., Sekar S. (2022). Front. Microbiol..

[cit51] Ye S., Liu F., Wang J., Wang H., Zhang M. (2012). Carbohydr. Polym..

[cit52] El-Newary S. A., Ibrahim A. Y., Asker M. S., Mahmoud M. G., El Awady M. E. (2017). Asian Pac. J. Trop. Med..

[cit53] Selim M., Amer S., Mohamed S., Mounier M., Rifaat H. (2018). J. Genet. Eng. Biotechnol..

[cit54] Ofoedu C. E., You L., Osuji C. M., Iwouno J. O., Kabuo N. O., Ojukwu M., Agunwah I. M., Chacha J. S., Muobike O. P., Agunbiade A. O., Sardo G., Bono G., Okpala C. O. R., Korzeniowska M. (2021). Foods.

[cit55] Guo R., Chen M., Ding Y., Yang P., Wang M., Zhang H., He Y., Ma H. (2022). Front. Nutr..

[cit56] Shi L. (2016). Int. J. Biol. Macromol..

[cit57] Abdel-Wahab B. A., Abd El-Kareem H. F., Alzamami A., Fahmy C. A., Elesawy B. H., Mostafa Mahmoud M., Ghareeb A., El Askary A., Abo Nahas H. H., Attallah N. G. M., Altwaijry N., Saied E. M. (2022). Metabolites.

[cit58] He Y., Ye M., Jing L., Du Z., Surhio M. M., Xu H., Li J. (2015). Carbohydr. Polym..

[cit59] Tahmourespour A., Ahmadi A., Fesharaki M. (2020). Int. J. Biol. Macromol..

[cit60] Śliżewska K., Markowiak-Kopeć P., Śliżewska W. (2020). Cancers.

[cit61] Wu J. (2021). Carbohydr. Polym..

[cit62] Li S., Shah N. P. (2016). J. Food Sci..

[cit63] Gangalla R., Macha B., Kasarla S., Eerla R., Thampu R. K. (2018). Int. J. Pharm. Biol. Sci..

[cit64] Jenab A., Roghanian R., Emtiazi G. (2020). Drug Des., Dev. Ther..

[cit65] Li M., van Esch B. C. A. M., Wagenaar G. T. M., Garssen J., Folkerts G., Henricks P. A. J. (2018). Eur. J. Pharmacol..

[cit66] Soria-Mercado I. E. , Villarreal-Gómez L. J., Rivas G. G. and Sánchez N. E. A., Bioactive Compounds from Bacteria Associated to Marine Algae, IntechOpen, 2012

[cit67] Das U. N. (2012). Ann. Hepatol..

[cit68] Rossner S., Ueberham U., Schliebs R., Perez-Polo J. R., Bigl V. (1998). Prog. Neurobiol..

[cit69] Čolović M. B., Krstić D. Z., Lazarević-Pašti T. D., Bondžić A. M., Vasić V. M. (2013). Curr. Neuropharmacol..

[cit70] Eldufani J., Blaise G. (2019). Alzheimer's Dement.: Transl. Res. Clin. Interv..

[cit71] Mehta M., Adem A., Sabbagh M. (2011). Am. J. Alzheimer's Dis..

[cit72] Hashimoto M., Kazui H., Matsumoto K., Nakano Y., Yasuda M., Mori E. (2005). Am. J. Psychiatry.

[cit73] Yurchenko E. A., Menchinskaya E. S., Pislyagin E. A., Trinh P. T. H., Ivanets E. V., Smetanina O. F., Yurchenko A. N. (2018). Mar. Drugs.

[cit74] Woodling N. S., Colas D., Wang Q., Minhas P., Panchal M., Liang X., Mhatre S. D., Brown H., Ko N., Zagol-Ikapitte I., van der Hart M., Khroyan T. V., Chuluun B., Priyam P. G., Milne G. L., Rassoulpour A., Boutaud O., Manning-Boğ A. B., Heller H. C., Andreasson K. I. (2016). Brain.

[cit75] Moore A. H., Bigbee M. J., Boynton G. E., Wakeham C. M., Rosenheim H. M., Staral C. J., Morrissey J. L., Hund A. K. (2010). Pharmaceuticals.

[cit76] Gangalla R., Gattu S., Palaniappan S., Ahamed M., Macha B., Thampu R. K., Fais A., Cincotti A., Gatto G., Dama M., Kumar A. (2021). Polymers.

[cit77] Almasi F., Mohammadipanah F., Adhami H.-R., Hamedi J. (2018). J. Appl. Microbiol..

[cit78] Drukarch B., Schepens E., Stoof J. C., Langeveld C. H., Van Muiswinkel F. L. (1998). Free Radical Biol. Med..

[cit79] Dehhaghi M., Tan V., Heng B., Mohammadipanah F., Guillemin G. J. (2019). Neuroscience.

[cit80] Mohd Nadzir M., Nurhayati R. W., Idris F. N., Nguyen M. H. (2021). Polymers.

