# Supplementary material for: In vitro studies on the pharmacological potential, anti-tumor, antimicrobial, and acetylcholinesterase inhibitory activity of marine-derived Bacillus velezensis AG6 exopolysaccharide
Source: RSC Adv. 2023 Sep 4;13(38):26406–17. doi: 10.1039/d3ra04009g (PMC10476021; doi:10.1039/d3ra04009g)
Supplement: RA-013-D3RA04009G-s001 [file RA-013-D3RA04009G-s001.pdf]

Electronic Supplementary Material (ESI) for RSC Advances.
This journal is © The Royal Society of Chemistry 2023

# Supplementary Data

## In-vitro studies on the pharmacological potential, anti-tumor, antimicrobial, and acetylcholinesterase inhibitory activity of marine-derived *Bacillus velezensis* AG6 exopolysaccharide

**Table S1.** Culture and Morphological Characteristics of *Bacillus velezensis* strain AG6

| *Bacillus velezensis* | Shape and Culture Characteristics |
|---|---|
| Strain AG6 | Gram postive Rod, irregular large colony, pale yellow, Rough Colony texture, Dull Colony surface, Convex, , Flat Elevation, non-capsulated, spore forming, non-motile, non-acid fast |

**Table S2.** Physiological and biochemical Characteristics of *Bacillus velezensis* strain AG6.

| Physiological characteristics | *Bacillus velezensis* strain AG6 |
|---|---|
| Catalase | + |
| Citrate | + |
| Coagulase | - |
| Indol | - |
| MR(Methylred) | - |
| VP (Voges proskauer) | + |
| Oxidase | - |
| $H_2S$ | - |
| Urease | + |
| Nitrate reduction | + |
| Glucose | + |
| Lactose | + |
| Sucrose | - |
| Mannose | - |

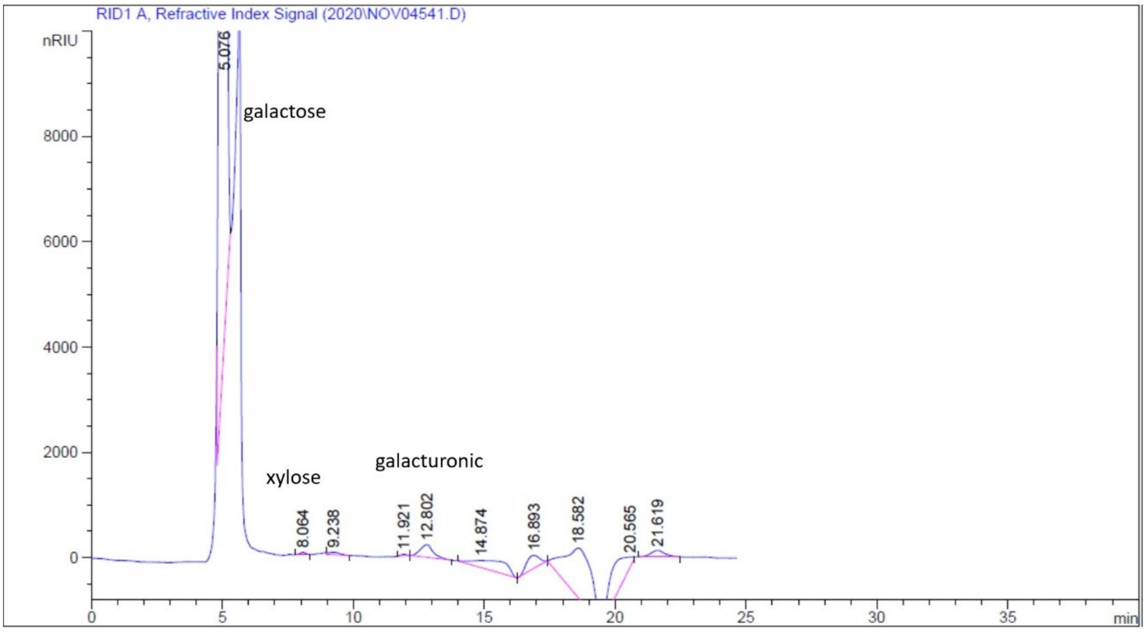

**Figure S1.** HPLC analysis of the EPSF6 from *Bacillus velezensis* strain AG6

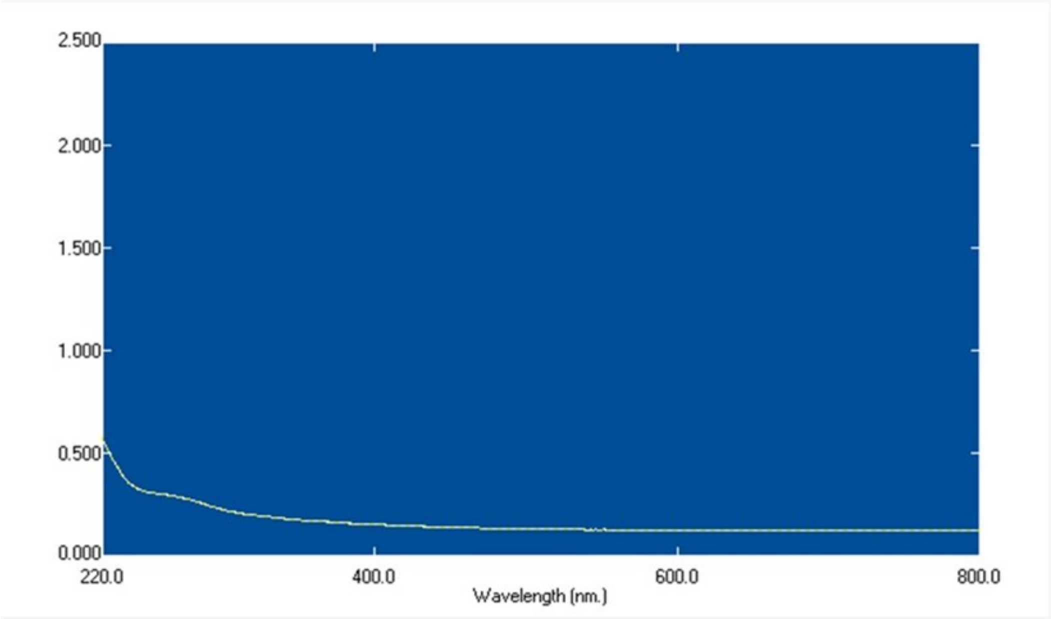

**Figure S2.** U.V Spectrum of EPSF6 isolated from *Bacillus velezensis* strain AG6.

**Antioxidant Control (Ascorbic Acid)**

**Table S3.** The ability of ascorbic acid to scavenge DPPH free radicals over different time intervals

| Concentration (μg/mL) | DPPH | $H_2O_2$ | ABTS |
|---|---|---|---|
| 20 | 61.47±1.02 | 55.78±1.12 | 64.96±1.24 |
| 40 | 82.16±1.92 | 70.34±1.73 | 83.64±1.11 |
| 60 | 91.59±1.10 | 84.62±1.13 | 90.27±0.83 |
| 80 | 97.10±0.87 | 92.40±0.96 | 95.68±1.64 |
| 100 | 99.13±1.45 | 97.58±1.05 | 99.14±1.39 |
| $IC_{50}$ | 86.44±1.42 | 88.71±0.98 | 87.50±0.75 |

**Antitumor Control (Cisplatin)**

**Table S4.** Cisplatin $IC_{50}$ against different cancerous cell lines

| Cell line | Cisplatin $IC_{50}$ (μg/ml) | S.D. (±) |
|---|---|---|
| HepG-2 | 1.29 | 0.17 |
| A-549 | 4.08 | 0.46 |
| HCT-116 | 2.36 | 0.32 |
| HEp-2 | 4.21 | 0.19 |
| PC-3 | 3.79 | 0.35 |
| MCF-7 | 3.41 | 0.25 |

**Anti-inflammatory 5-LOX control (ibuprofen)**

**Table S5.** The inhibitory activity of ibuprofen against 5-LOX enzymatic activity.

| conc. (μg/ ml) | Mean of LOX inhibitory% |
|---|---|
| 125 | 100.00 |
| 62.5 | 100.00 |
| 31.25 | 100.00 |
| 15.63 | 89.21 ±1.2 |
| 7.81 | 78.34 ±0.92 |
| 3.9 | 70.12 ±2.1 |
| 1.95 | 57.32 ±1.3 |
| 0.98 | 42.16 ±0.63 |
| 0 | 0 |
| $IC_{50}$ | 1.5±1.3 |

**Anti-inflammatory COX-2 control (celecoxib)**

**Table S6.** The inhibitory activity of celecoxib against COX-2 enzymatic activity.

| conc. (µg/ ml) | Mean of LOX inhibitory% |
|---|---|
| 31.25 | 100 |
| 15.63 | 100 |
| 7.81 | 100 |
| 3.9 | 100 |
| 1.95 | 82.15 |
| 0.98 | 66.34 |
| 0.49 | 52.72 |
| 0.24 | 49.35 |
| 0.12 | 19.35 |
| 0 | 0.00 |
| $IC_{50}$ | 0.28± 1.7 |

**Table S7.** The inhibitory activity of Eserine against acetylcholinesterase activity.

| Conc. (µg/ ml) | Activity (%) |
|---|---|
| 0.02 | 15.09 ± 1.02 |
| 0.04 | 26.67 ± 1.36 |
| 0.06 | 37.11 ± 1.15 |
| 0.08 | 45.92 ± 1.08 |
| 0.1 | 55.36 ± 1.14 |
| 0.12 | 64.78 ± 1.20 |
| $IC_{50}$ | 0.09(µg/ ml) |
